# Acute Toxicity and Dermal and Eye Irritation of the Aqueous and Hydroalcoholic Extracts of the Seeds of “Zapote” *Pouteria mammosa* (L.) Cronquist

**DOI:** 10.1155/2015/642906

**Published:** 2015-07-26

**Authors:** Carlos M. S. Dutok, Clara Azalea Berenguer-Rivas, Elizabeth Rodríguez-Leblanch, Liliana Pérez-Jackson, Idelsy Chil-Nuñez, Julio César Escalona-Arranz, Bernardo Reyes-Tur, Margareth M. C. Queiroz

**Affiliations:** ^1^Laboratório de Entomologia Médica e Forense, Instituto Oswaldo Cruz-Fundação Oswaldo Cruz (IOC/FIOCRUZ), Avenida Brasil, 4365 Pavilhão Herman Lent, Sala 14, Térreo, Manguinhos, 21040-900 Rio de Janeiro, RJ, Brazil; ^2^Programa de Pós-Graduação em Biodiversidade e Saúde, Bolsista com Financiamento CAPES/MES-Cuba Projeto 130/11, Instituto Oswaldo Cruz-Fundação Oswaldo Cruz (IOC/FIOCRUZ), Avenida Brasil 4365, Secretaria Académica, Pavilhão Arthur Neiva, Manguinhos, 21040-900 Rio de Janeiro, RJ, Brazil; ^3^Departamento de Farmacia, Facultad de Ciencias Naturales, Universidad de Oriente, Patricio Lumumba y Avenida de Las Américas, 90500 Santiago de Cuba, Cuba; ^4^Centro de Toxicología y Biomedicina, Universidad de Ciencias Médicas de Santiago de Cuba, Autopista Nacional Km. 1.5, Apartado 4033, 90400 Santiago de Cuba, Cuba; ^5^Departamento de Biología, Facultad de Ciencias Naturales, Universidad de Oriente, Patricio Lumumba y Avenida de Las Américas, 90500 Santiago de Cuba, Cuba

## Abstract

The common use of *Pouteria mammosa* (L.) Cronquist, “Mamey or Zapote,” in food and ethnobotanic medicine shows its low or absent toxicity as fruit extracts prepared from seeds. However, it is essential to conduct security trials to scientifically support their use in drug therapy. This study evaluated the aqueous and hydroalcoholic extract (25%) Acute Oral Toxicity, obtained from the seeds of *P. mammosa*, in Sprague Dawley rats and dermal and eye irritability in New Zealand rabbits. The 404 and 405 acute dermal and eye irritation/corrosion guidelines were used, as well as the 423 Acute Oral Toxicity guideline, Acute Toxic Class Method of the Organization for Economic Cooperation and Development (OECD). The aqueous extract was located in the following category: not classified as toxic (CTA 5), while hydroalcoholic extract at 25% was classified as dangerous (CTA 4). Both extracts can be used without side reaction that irritates the skin which permitted classification as potentially not irritant. *P. mammosa* in the two extracts caused mild and reversible eye irritation, and it was classified as slightly irritating.

## 1. Introduction

It is believed that the word “Zapote” has as origin the Aztec word “tzapotl,” which was generally applied to all sweet and soft fruits. For a long time this has been the common name for* Pouteria mammosa* (L.) Cronquist. This plant fruit has been traditionally used for its medicinal properties against fever, inflammation, skin rashes, ulcers, nausea, vomiting, and diabetes, besides being a rich source of nutrients [1]. In Cuba, the milky bark juice has been used since at least 1864 to destroy warts, while the extract of the seeds is used as emollient in painful skin diseases [2]. Also, infusions of the seed are used to treat cough and bronchitis, not only in Cuba but also in Costa Rica [3].

Species from the Sapotaceae family have been well studied for their insecticidal and larvicidal actions such as the case of* Pouteria venosa* (Mart.) Baehni. In this species, four isolated triterpenes (taraxerol, ursolic acid, 3*β*,19*α*,23-trihydroxyurs-12-en-28-oic acid, and 2*α*,3*α*,19*α*,23-tetrahydroxyurs-12-en-28-oic acid) and a phytosteroid (spinasterol) were active against 4th instar larvae of* Aedes aegypti* [4]. In wood and bark extracts of* Pouteria guianensis* Aubl. chemical compounds with repellent properties against* Nasutitermes* sp. were identified [5]. Pouterin, a lectin-like protein isolated from* Pouteria torta* (Mart.) Radlk. seeds, caused 50% mortality in larvae of the insect* Callosobruchus maculatus* F. (Coleoptera) when incorporated in the diet [6]. It is also known that this protein presents an insecticidal effect against* Anagasta kuehniella* (Lepidoptera: Pyralidae) larvae [7]. These insects are recognized as a major cause of loss in stored grains throughout the world. Recently the activity of* Pouteria mammosa* (L.) Cronquist aqueous extract in the postembryonic development of the blowfly* Chrysomya putoria* (Calliphoridae) was evaluated [8]. The results revealed a 47.5% decrease in the viability of the flies. The production of myiasis by flies and its affinity with humans and domestic animals has been known since antiquity [9, 10]. These results place* P. mammosa* as a candidate for alternative insect control, which is currently performed almost exclusively with the use of organophosphate insecticides, which are toxic to living beings, can cause serious environmental damage, and may induce or develop resistant insects [11, 12].

The ancient and common use of “Zapote” as food and/or therapeutic alternative signs it as not dangerous for human health. However, to scientifically support its use in drug therapy, it is essential to accomplish trials attesting its security. This study therefore evaluated the Acute Oral Toxicity and the Dermal and Eye Irritability tests of the aqueous and hydroalcoholic extracts (25%), obtained from the seeds of* P. mammosa*.

## 2. Materials and Methods

### 2.1. Plant Material

*Pouteria mammosa* fruits were collected in February of 2012 in the town of “El Caney” Santiago de Cuba. Leaves and seeds were taxonomically identified by specialists of “Centro Oriental de Ecosistemas y Biodiversidad (BIOECO)” from the Natural History Museum Tomas Romay—Santiago de Cuba City. Desiccated specimens were deposited into the herbarium of the same institution under the registration number Hac 468.

### 2.2. Zapote Seeds Extracts

The endocarp from seeds of* P. mammosa* was removed and the endosperm pulverized in a knife mill. Aqueous and hydroalcoholic (25%) extracts in the proportion of 40 grams in 100 milliliters were prepared by maceration with agitation in a sieve for 8 hours. The extracts were filtered, bottled in amber flasks, and stored at 8°C. A qualitative phytochemical screening was carried out to determine the presence of alkaloids, triterpenes and/or steroids, quinones, coumarins, lipid and/or essential oils, mucilage, saponins, phenols and/or tannins, amino acids, reducing sugars, cardiac glycosides, flavonoids, cyanogenic glycosides, and resins [13].

### 2.3. Animals and Ethical Considerations

All the animals included in the study received during their lifetime water and food* ad libitum*. They were maintained under favorable environmental conditions with a temperature of 25°C, relative humidity between 40 and 70%, and cycles of light and darkness of 12/12 hours. Experiments were carried out following ethical guidelines towards animals and on the established principles of Reduction and Refinement. For Acute Oral Toxicity tests, six nulliparous female Sprague Dawley rats were used, aged between 5 and 6 weeks and weighing between 170 and 206 grams, provided by the National Center for Laboratory Animal Production (CENPALAB/Health Certificate number 08001414). Tests for Dermal and Eye Irritability were based, in each case, on three New Zealand line rabbits with ages of 11 ± 1 weeks and weight from 2 to 3 kg. Rabbits were provided by the Center for Reproduction of Rabbits “El Modelo” of Santiago de Cuba (Health Certificated number 0314).

### 2.4. Acute Toxicity Test

The* Guidelines for Testing of Chemicals*, Acute Oral Toxicity-Acute Toxic Class Method 423 of the Organization for Economic Cooperation and Development (OECD), was used [17]. Substances ranges are set in toxic classes of not classified, dangerous, toxic, very toxic, and highly toxic as shown in Table 1.

Food was suspended 12 hours before starting the study and the body mass was determined moments before the administration of the extract. Animals were randomly assigned to two groups of three rats each: a control group treated with physiological saline and another group treated with the extracts. To the first experimental group a dose of 300 mg/kg was given using an orogastric tube. Clinical observations of animals were performed four times per day, paying attention to behavior, general physical condition, nasal mucosa, changes in skin and fur, respiratory frequency, somatomotor activity, and possible occurrence of signs such as tremors, convulsions, diarrhea, lethargy, drooling, low response to stimuli, sleep, photophobia, and coma. Palpation of the abdomen was carried out as well. After 48 hours of clinical observation without any signs of toxicity, a second experimental group was carried out administering 2000 mg/kg of extract. Animals were weighed on the seventh and fourteenth days. The animals were humanely euthanized at the end of the study by administering an overdose of the anesthetic ketamine intraperitoneally. Internal organs were subsequently studied macroscopically.

### 2.5. Dermal Irritation Test

Animals were shaved 24 hours before the application of the extracts on the back and both flanks (10% body surface). Skin was washed with sterile water and allowed to stand for 24 hours. Patches with 0.5 mL of extract were applied on an area of about 6 cm^2^ of one flank, and the other flank was used as a reference. Animals were exposed to the extract for four hours after which the patches were removed and the application area was washed with sterile water. Observations were recorded at 1, 24, 48, and 72 hours after the removal of the patches. Behavior, general condition, posture and reflexes, attitude towards food, water, and hygiene were evaluated. Weights were recorded and compared at the beginning and the end of the study. Evaluation of edema and erythema was performed and the* Dermal Irritation Score* (DIS) was calculated by the following formula:

(1) $$\begin{array}{c} \text{DIS}=\frac{\text{Value }\left(\text{erythema}+\text{edema}\right)}{\text{Nr. of animals}\times \text{Nr. of observations}}. \end{array}$$

The extracts were classified as proposed by Draize et al., 1944, scale [15] (Table 2) and under the guidance for the evaluation of chemicals issued by the Organization for Economic Cooperation and Development, methodology used for determining the degree of acute dermal irritation/corrosion, 404 [17].

### 2.6. Eye Irritation Test

The guide for the evaluation of chemical substances issued by the Organization for Economic Cooperation and Development was used, following the methodology for determining the degree of acute eye irritation/corrosion, 405 [18]. A total of three rabbits per test group were subjected to a rigorous study of the ocular structures: cornea, iris, and conjunctiva. A volume of 0.1 mL of extract was instilled to the bottom of the right conjunctival sac, keeping eyelids together over the next 20 minutes. Both eyes of each animal were examined at the time and 24, 48, and 72 hours after, always by the same specialist. Corneal damage was determined in a dark room with the use of a solution of 2% sodium fluorescein, and physiological saline was used to remove excess solution from the instilled developer substance. Finally an ultraviolet light was used for observation. Observations were made up to five days to assess reversibility of the effects, and animals were weighed at the end of the study to compare variations in this parameter. The* Ocular Irritation Score* (OIS) was determined using the formula below [18]:

(2) $$\begin{array}{c} \text{OIS}=\sum \frac{\text{Individual observations}}{\text{Nr. animals}\times \text{Nr. observations}}. \end{array}$$

The value obtained was compared with the ranges defined in Table 3 to give the results of approval or rejection, at the discretion of the Cuban method proposed by García-Simón et al., 1988, defined as OIS approval limits from 0 to 19 and rejection from 20 to 110 [16].

### 2.7. Statistical Analyses

Results were presented as means of at least three replications. Unpaired “*t*” test with Welch's correction for body weight comparisons was performed using GraphPad Prism version 5.00 for Windows, GraphPad Software, San Diego, California, USA.

## 3. Results and Discussion

### 3.1. Phytochemical Screening

It was determined that both extracts (aqueous and hydroalcoholic at 25%) contained, in similar intensities, coumarins, saponins, phenols, and tannins, suggesting similar amounts. More flavonoids and cyanogenic glycosides were present in the aqueous extract than in the hydroalcoholic one. None of the extracts showed the presence of resins, cardiotonic glycosides, and mucilages. Metabolites which caused most differences between the two extracts were lipids and/or essential oils, amino acids, and reducing sugars that are only contained in the aqueous extract. Alkaloids, quinones and triterpenes, and steroids were evident only in the hydroalcoholic extract at 25% of* P. mammosa*.

### 3.2. Acute Toxicity

The employed doses of the aqueous extract of* P. mammosa* (2000 mg/kg) did not cause significant changes in the clinical signs in rats within 24 to 72 hours. After this period one of the animals showed little response to stimuli and subsequently died. The other two rats were subjected to a strict observation and clinical assessment within 14 days of the study, not presenting any alteration or irregularity in clinical signs. In the case of the hydroalcoholic extract at 25%, the first dose level (300 mg/kg) did not result in significant changes in clinical signs in rats. At 48 hours, the highest dose (2000 mg/kg) was administered. Three animals died. One rat died at 6 hours having presented cyanosis, little response to stimuli, and loose stools before death. Two other rats died at 7 and 12 hours, showing similar clinical signs of rectal bleeding. At necropsy, the animals showed no macroscopic alterations of the organs except in the case of the dead rat at the dose level of 2000 mg/kg of the aqueous extract of* P. mammosa*, whose stomach showed an increase in size caused by gases. Intestinal edema with fibrinous exudate and distended cecum was also observed, which can be found related to the presence of cyanogenic glycosides contained in the extract.

According to Alemán and Gad et al., body weight is often the most sensible indicator of an adverse effect [19–21]. Other authors, such as Mosberg and Hayes, have argued that the data referring to body weight have a high sensitivity to alterations due to chemicals with low toxicity [22]. It is considered that among the indicators that provide more information in toxicological studies is the rapid loss of body weight (approximately 15 to 29% loss of body weight in a period of five to seven days) [23, 24].

**Figure 1:**
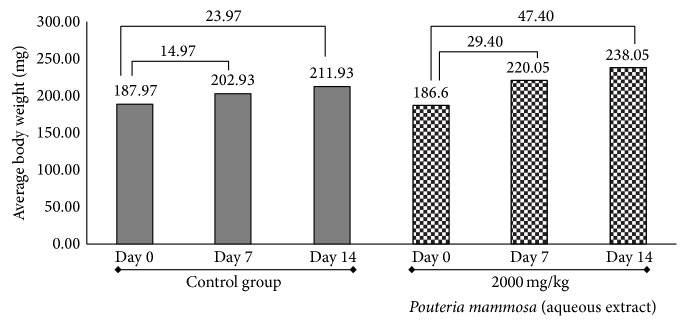
Behavior of body weight of rats in the Acute Oral Toxicity test of aqueous extract of the seeds of* Pouteria mammosa* (L.) Cronquist (Zapote). Numbers on the horizontal lines represent weight gain in milligrams (mg) from day 0 until the seventh and final day of the trial.

**Figure 2:**
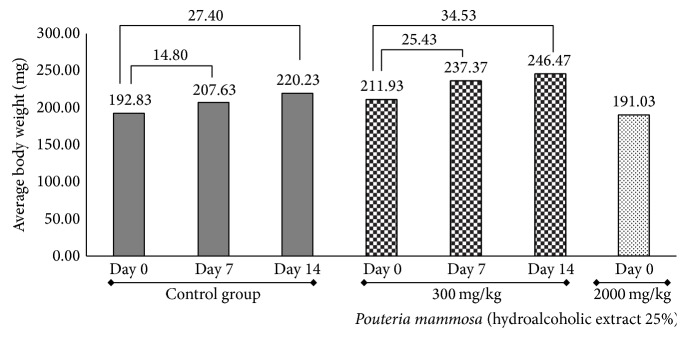
Behavior of the body weight of rats in Acute Oral Toxicity test of hydroalcoholic extract at 25% of seeds of* Pouteria mammosa* (L.) Cronquist (Zapote). Numbers on the horizontal lines represent weight gain in milligrams (mg) from day 0 until the seventh and final day of the trial.

It was observed that the body weight was not affected after administration of the extracts (Figures 1 and 2), showing a normal increase, which corresponds to standard references to the use and care of laboratory animals, in relation to the species used [23, 25]. When the aqueous extract was analyzed (Figure 1), in the case of the group treated with* P. mammosa*, weight gain was 47.40 g (21.61%) and an increase in weight of 23.97 g (representing 11.31%) was observed in the control group. These results were corroborated by statistical analysis and significant differences, *p* < 0.05, in the unpaired “*t*” test were found with Welch's correction. The treated group assimilated the maximum dose of 2000 mg/kg.

The hydroalcoholic extract of* P. mammosa* at 25% proved to have a superior level of toxicity because the maximum dose that was assimilated by the rats was 300 mg/kg (Figure 2). The control group had a 27.40 g increase until the end of study representing a 12.44% weight gain and treated group had an average increase of 34.53 g representing a 14.01% weight gain. No significant differences were found between the treated and the control groups when performing statistical analysis. When the 2000 mg/kg dose of hydroalcoholic extract of* P. mammosa* at 25% was administered all animals died. Weight gain was evident in all animals included in the study and was greater in those treated with extracts of* P. mammosa* in both forms, aqueous and hydroalcoholic 25%. This effect found for body weight is consistent with results obtained by Petit et al.; Sharma et al.; Rao et al.; and Elmnan et al. that studied the effect of fenugreek seeds over metabolism of rats (*Trigonella foenum-graecum *L.). They reported that saponins increased food consumption, thus resulting in increased weight gain in rats [26–29]. In phytochemical screening of* P. mammosa* it was determined that both extracts (aqueous and hydroalcoholic at 25%) contained saponins, in similar intensities, which could justify the same effect in this study. Coincidentally a similar result was obtained in invertebrate organisms by Carriço et al. that studied the effect of* P. mammosa* on immature stages of* Chrysomya putoria*, wherein the groups treated with the aqueous extract of leaves resulted in an increase in body weight of maggots [8].

The group treated with 25% hydroalcoholic extract of* P. mammosa* showed the same evidence for the three dead rats at the dose level used: distended stomach with bloody walls, edematous and hemorrhagic bowel, and cyanotic walls of the stomach and intestinal mucosa. These results could be expected considering the abundance of alkaloids observed in the chemical composition of the hydroalcoholic extract, shown by the phytochemical screening. Some alkaloids of plant substances can act on cholinergic receptors at some neuroeffector junctions (acting as cholinomimetic agent) and the myenteric plexus in the gastrointestinal tract (GI) significantly stimulating the digestive tract. They can also induce tracheobronchial secretions and stimulate bronchial smooth muscle, resulting in intense bronchoconstriction and reduced vital capacity [30].

### 3.3. Dermal Irritation

Rabbits showed no signs of irritation or skin edema. The skin was intact when patches were removed and within 72 hours of the study. The Dermal Irritation Score for aqueous and hydroalcoholic 25% extracts of seedsof* P. mammosa* was equal to “0.” Body weight in rabbits was not affected in any case after the application of extracts. A normal increase of body weight was observed corresponding to the established rules for handling laboratory animals, in relation to the species [23].

### 3.4. Eye Irritation

Ocular Irritation Score was 10.5 in the aqueous and slightly higher in the hydroalcoholic 25% extract of* P. mammosa* (OIS = 15.3). The index for both cases is within acceptable limits as set by García-Simón et al. [16]. It was evident that the effects on the eye were reversible within 96 hours for those that occurred in the conjunctiva, 72 hours for those that occurred in the iris, and 48 hours for those on the cornea (Table 4). The behavior of body weight in rabbits was not affected after administration of the extracts. No clinical signs or changes in the behavior of animals associated with the administration of the extracts were evident.

## 4. Conclusions

Aqueous extract of* Pouteria mammosa* (L.) Cronquist was located in the following category: not classified as toxic (CTA 5), established by the guideline 423 of the Organization for Economic Cooperation and Development (OECD). However, due to the occurrence of death of an animal and the manifestation of several clinical signs, the assessment of the subchronic toxicity is suggested by a toxicity test repeated dose for 28 days. The hydroalcoholic extract 25% at the dose of 2000 mg/kg showed clinical signs of toxicity and death of all animals, gross lesions in organs and organ systems. The extract was classified as dangerous (CTA 4), with a LD_50_ mortality range between 300 and 2000 mg/kg. Neither extract induced any apparent adverse clinical signs in animals when applied on the skin, showing that* P. mammosa* can be used without side reaction on organs. Both extracts were classified as potentially not irritant, according to guideline 404 of the Organization for Economic Cooperation and Development (OECD) which evaluates acute dermal irritation/corrosion. Both extracts obtained from seeds of* P. mammosa* caused mild and reversible damage. They were classified as slightly irritant, according to the guideline 405 of the Organization for Economic Cooperation and Development (OECD) which evaluates acute eye irritation/corrosion.

## Figures and Tables

**Table 1:** Classification of substances according to the guideline 423 of the Organization for Economic Cooperation and Development [14].

DL_50_ ranges (mg/kg)	ATC	Classification
DL_50_ > 2000 mg/kg	ATC 5	Not classified
300 < DL_50_ ≤ 2000 mg/kg	ATC 4	Dangerous
50 < DL_50 _≤ 300 mg/kg	ATC 3	Toxic
5 < DL_50_ ≤ 50 mg/kg	ATC 2	Very toxic
DL_50_ < 5 mg/kg	ATC 1	Highly toxic

**Table 2:** Dermal Irritation Scores ranges established by Draize et al. 1944 [15] for classification of substances irritating effect on the skin.

Ranges of Dermal Irritation Score	Classification of dermal irritability
0 < DIS < 0.4	Not irritant
0.4 ≤ DIS < 2.0	Slightly irritating
2.0 ≤ DIS < 5.0	Moderately irritating
5.0 ≤ DIS ≤ 8.0	Severely irritating

**Table 3:** Ocular Irritation Scores ranges established under the Cuban method for classification of eye irritation/corrosion [16].

Ranges of Ocular Irritation Score	Classification of eye irritability
0 < OIS < 10	Not irritant
10 ≤ OIS < 20	Slightly irritating
20 ≤ OIS < 30	Moderately irritating
30 ≤ OIS ≤ 110	Severely irritating

**Table 4:** Evaluation of ophthalmic damage caused by the aqueous and hydroalcoholic 25% extracts of seeds of *Pouteria mammosa* (L.) Cronquist (Zapote).

Hours	Damage observed in the group treated with aqueous extract of seeds of *Pouteria mammosa *			Damage observed in the group treated with hydroalcoholic 25% extract of seeds of *Pouteria mammosa *		
	Conjunctiva	Iris	Cornea	Conjunctiva	Iris	Cornea
1	34	20	40	26	20	110
24	18	10	10	24	10	20
48	12	5	0	12	0	0
72	8	0	0	8	0	0
96	0	0	0	0	0	0
Total	72	35	50	70	30	130

Number of observations	157			230		
Ocular Irritation Score	10.5			15.3		
